# PROTOCOL: Do hospital leadership styles predict patient safety indicators? A systematic review

**DOI:** 10.1002/cl2.1338

**Published:** 2023-07-07

**Authors:** Sandra C. Buttigieg, Nicoletta Riva, Gianpaolo Tomaselli, Emanuel Said, Elaine Grech, Vincent Cassar

**Affiliations:** ^1^ Department of Health Systems Management and Leadership, Faculty of Health Sciences University of Malta Msida Malta; ^2^ Department of Pathology, Faculty of Medicine and Surgery University of Malta Msida Malta; ^3^ Department of Marketing, Faculty of Economics, Management and Accountancy University of Malta Msida Malta; ^4^ Department of Business and Enterprise Management, Faculty of Economics Management and Accountancy University of Malta Msida Malta

## Abstract

This is the protocol for a Campbell systematic review. The objectives are as follows: The main aim of this systematic review is to identify whether hospital leadership styles predict patient safety as measured through several indicators over time. The second aim is to assess the extent to which the prediction of hospital leadership styles on patient safety indicators varies as a function of the leader's hierarchy level in the organization.

## 1 BACKGROUND

### 1.1 Description of the condition

Recent healthcare scandals worldwide have called attention to the role of hospital leadership. A case in point is the Mid Staffordshire UK NHS Foundation Trust scandal involving deaths of hundreds of patients between 2005 and 2008 due to appalling failings in care, eloquently reported in the Francis Inquiry (Francis, 2013). This scandal raised troubling questions about hospital leadership and its influence on the professional standards practiced. The main recommendation of this Inquiry was to build stronger health care leadership, to promote compassionate and committed care while ensuring openness, transparency and candor. Despite specific actionable recommendations, more scandals continue. In a more recent scandal attributed to poor leadership, the American Veterans Administration VA Hospital in Phoenix was reported to provide delayed care to patients, causing the death of 43% of 225 patients between October 2014 and August 2015 (CBS News, 2017).

The term “leader” tracks back to around 1300 A.D., but the term “leadership” emerged in 19th century literature (Antonakis & Day, 2018). Since then, leadership has evolved and shifted through various theories and theoretical models across the 20th century and into the 21st century. Our review focuses specifically on leadership in the hospital context. We consider the notion of”hospital leader” to be nested within the broader concept of “hospital leadership,” which incorporates the roles of one or more individuals and sets of behaviors that elicit reactions of others in the setting that culminate in desirable quality of care delivery, reflected in this case by patient safety (Allio, 2012; Day et al., 2014; Fulop & Day, 2010). We treat hospital leadership as an organizational phenomenon rather than an individual one. It encompasses the people holding the title, the followers and the exchanges that occur in specific contexts. In addition, we allow this interactive process between people and context to include both clinicians and non‐clinicians insofar that such processes impact patient safety. Hospital leadership assumes a key role in inspiring patient safety cultures and strategies (DiCuccio, 2015).

Although culture and climate are often used interchangeably, safety climate is the measurable component of safety culture (Colla et al., 2005), noting that “One particular focus is the evaluation of “safety climate,” a term that generally refers to the measurable components of “safety culture” such as management behaviors, safety systems, and employee perceptions of safety” (2005, p. 364). Therefore, in this protocol, we will adhere to the facets/indicators of safety climate (McFadden et al., 2014; Merrill, 2015) as most recently listed by a team of experts on patient safety as part of the ERNST—COST Action namely: communication about error, communication openness, organizational learning, patient safety rating, response to error, staffing, supervisor and management support, teamwork and work pressure and pace.

Hospital leadership involves both clinical, namely physicians, surgeons, and nurses among others, as well as non‐clinical administrative and technical professional staff. Evidence suggests that patient safety needs to be improved in hospitals through leadership that is able to respond to patient safety issues (Francis, 2013). Francis (2013) emphasizes, “It was not a single rogue healthcare professional who delivered poor care in Stafford, or a single manager who ignored patient safety, who caused the extensive failure which has been identified. There was a combination of factors, of deficiencies throughout the complexity that is the NHS” (p.36, Executive Summary). Indeed, Francis (2013) provides a leadership framework with specific focus on patient safety (c.f. recommendation 216, Vol. 3, p. 1593).

Leadership styles that focus on people and relationships are crucial to maximize patient safety (Wong & Giallonardo, 2013). They embrace relational processes enabling people to accomplish objectives, or benefit the common good (Clarke, 2018). Leadership style is defined by Nicolaou‐Smokoviti (Nicolaou‐Smokoviti, 2004) as “a stable mode of behavior that the leader uses in his or her effort to increase his or her influence, which constitutes the essence of leadership”. This definition is in line with Antonakis' discussion on “correctly modeling leadership style” ((Antonakis & Day, 2018), p. 70).

The Institute of Medicine (IOM) defines patient safety as the prevention of harm to patients implying a broad focus on a system of care delivery aiming at preventing errors, learning from existing errors, and building on a [climate] of safety that involves health care professionals at various levels (1999, 2001).

This systematic review therefore aims to answer the following question:

1. Does hospital leadership predict patient safety indicators?
  Moreover, Flin and Yule (2004) showed that leaders’ roles and responsibilities vary depending on their hierarchical positions. Therefore, the second review question is:
2. Does the leader's place in the organizational hierarchy moderate the prediction of hospital leadership styles on patient safety indicators?

The proposed systematic review will scope the literature for workable definitions for “hospital leader” and “leadership” to achieve absolute clarity.

### 1.2 Description of the intervention

This systematic review focuses exclusively on the prediction of leadership in hospitals on patient safety indicators. Examples of leadership styles reported in hospital settings are transformational leadership (Frich et al., 2015), authentic leadership (Alilyyani et al., 2018) and distributed leadership (Martin et al., 2015).

Leadership across contexts, not least in hospitals, is a multifaceted phenomenon that may be viewed from diverse angles related to the individual, results, position, the purpose and the process (Grint & Smolovic, 2016). The importance leaders attach to team members and followers highlights the follower‐centricity of leadership styles (Antonakis & Day, 2018). Such styles are assessed through leaders’ self‐reported measures, surveys or questionnaires administered to followers, external raters’ evaluation, or 360‐degree feedback (Dussault et al., 2013; Garman et al., 2004; Halbesleben et al., 2013). In addition, experimental studies in leadership are emerging (Antonakis, 2017; Antonakis et al., 2014; Hughes et al., 2018). The intervention in this review will specifically include followers’ ratings of their hospital leadership.

### 1.3 How the intervention might work

A variety of leadership constructs exist in organizational research. Some share a relational dimension, including commitment to leading through values (transformation), and creating trusting relationships with followers (authentic). These leadership processes are intended to convey a sense of inspiration and positivity in their followers enhancing them to develop meaningful work relations. Others are less relational in nature, including transactional leadership, which is intended to reinforce follower behavior and generate a sense of exchange between what one achieves and what one can gain as reward. Considerable research on various forms of leadership reports that these styles have differential outcomes, at least in part based on whether theirs is a relational and values‐oriented focus or more task goal‐oriented. In addition, recently emerging forms of leadership such as adaptive and distributed leadership focus more on broad engagement in problem solving and responses to changing environments.

This systematic review considers how others’ evaluation of their leaders predicts their adherence to patient safety. We posit that leadership styles predict patient safety outcomes based on the extent to which they promote relational attachment of workers to the organization, foster trust through authenticity, or promote shared responsibility and adaptation to change. Alternatively those styles that operate through less relational processes, such as transactional or authoritarian leadership are not predicted to promote patient safety. Through recognition‐based perceptual processes, followers evaluate the degree of fit between observed patterns of behavior of their leader and their inner beliefs of what a leader is or should be (Lord & Mahler, 2002). How followers perceive the leader is likely to regulate their behavior in terms of intentions and purpose of set goals and this is most probably responsible for the quality work environment (Hamstra et al., 2014). Consequently, the standards of behavior that are created will reinforce or diminish the importance of specific work practices related to patient safety. When there is a fit between the observed patterns of behavior and one's personal subjective construct of leadership, the individual is recognized as a leader from the point of view of the follower. These implicit perceptions stress the importance of the leader‐member exchange relationship (Lord & Emrich, 2001) and therefore predict the quality of error management (patient safety indicators) elicited by followers, who counteract the possible causes of wrong behaviors. In so doing, they ensure a systematic way to reduce failures or to identify errors earlier on. Thus, leadership styles, as seen from the followers’ perspective, predict patient safety indicators because leaders exert a modeling function. The style of leadership is likely to foster a working environment for followers where they can accept responsibility for their own actions and errors, avoid blaming others for errors, and can take corrective action in time (Farnese et al., 2019). Different leadership styles may go about successfully solving a variety of problems in diverse yet effective ways (Bedell‐Avers et al., 2008). For example, transformational leaders create a climate of safety, encourage adoption of patient safety initiatives, and in due course, work towards achieving positive improvements in patient safety indicators (McFadden et al., 2009). Authentic leadership contributes to safer work environments for patients and staff through the emphasis on transparency, balanced processing, self‐awareness and high ethical standards (Wong & Giallonardo, 2013). Distributed leadership across the health system bring about improvements in patient care by having leaders widely engaging colleagues and stakeholders, thereby pluralizing responsibility (McKee et al., 2013; West et al., 2014). Adaptive leadership is important to address patient safety because changes that need to be introduced face adaptive challenges (Choong et al., 2017). Therefore, adaptive leaders engage people to decide which changes are needed, while preserving what is safe practice (Pronovost, 2011). The leadership styles are not mutually exclusive, since a leader might have a mixture of them.

While there is a well‐known correlation between leadership and safety (Vincent, 2011), the prediction of hospital leadership on patient safety indicators is best explained through leadership process models as part of the input‐process‐outcome logic (Fischer et al., 2017). We, therefore, have considered a logic model as a visual to be the basis for the factors to be included in the content extracted from each empirical study and which will help us identify variation in studies in the kinds of linkages they test between leadership style and patient safety outcomes.

These processes are reflected in the logic model on which the review is based. The components of the logic model are: *Input:* Leadership (excluding dyadic level leadership e.g. LMX and focusing specifically on work group and organizational level leadership); *Activities:* We consider (i) enabling physical and psychosocial work environments (ii) promoting team think (iii) enhancing individual competences (iv) clarifying tasks structures as actions to attain the desired output; *Output:* The cumulative effect of the activities gives rise to a specific *modus operandi* which defines the patient safety climate; *Outcomes:* This patient safety climate is expected to reduce errors and near misses (primary outcomes), as well as reduce patient harm and patient mortality (secondary outcomes); and *Impact:* The long‐term fundamental effect of these outcomes is improved patient safety.

We acknowledge the possibility that leadership may not always predict patient care indicators positively. Indeed, we allow the possibility that leadership styles may adversely predict these indicators (McDonald, 2014; Sfantou et al., 2017; Tourish, 2020). Moreover, we also recognize that the leadership process across the organizational hierarchy may potentially influence the hospital leadership‐patient safety relationship differently.

### 1.4 Why it is important to do this review

This systematic review is being conducted to establish relationships between hospital leadership styles and patient safety indicators, over and above what is known from individual studies and to assess the extent of variations across these studies. There is still a fragmented perspective of patient safety (Rivard et al., 2005), with literature focusing on a narrow set of aspects (e.g., medical errors and surgical procedural errors among others). Indeed, we observe a relative dearth of comprehensive conceptualization of patient safety within hospitals. Likewise, operationalization of patient safety is scant with current key indicators focusing on specific aspects. There are several reviews that link leadership to patient safety (e.g. Künzle et al., 2010; Parand et al., 2014; Ring & Fairchild, 2013). Given that a correlation is observed between leadership and hospital performance (Aiken et al., 2002; Institute of Medicine, 2001; Shipton et al., 2008), evaluating the extent of prediction is important. Equally, there is a need to understand how the leader's place as part of the leadership process in the organizational hierarchy can influence this relationship. The Francis report (Francis, 2013) among others has clearly shown that leadership or lack of it is a strong determinant of hospital effectiveness, with the latter being clearly manifested through patient safety. Likewise, OECD (2017) considers patient safety as a critical policy issue that calls for more leadership action. The OECD (2017) report estimates that about one in ten patients are harmed during health care. This translates into approximately 10% of hospitalizations suffering adverse events during these hospitalizations and that patient harm is the 14th leading cause of morbidity and mortality across the world. The financial cost of patient harm on health systems runs into trillions of dollars annually with the current evidence suggesting that 15% of hospital expenditure is attributed to treating safety failures. Because patient safety is such a core key performance indicator for hospitals, more than justifies studying the hospital leadership styles‐patient safety indicators relationships. The cost of errors and harm, with their consequent considerable waste of healthcare resources, can be prevented by meaningful investment in areas such as leadership. The same OECD report estimates that the cost of failures by far outweighs any investment that is needed to fulfill successful prevention.

## 2 OBJECTIVES

The primary objective of this systematic review is to investigate leadership styles’ prediction of patient safety indicators.

The secondary objective is to examine the extent to which the hierarchical level of the leader moderates the prediction of leadership styles on patient safety indicators.

## 3 METHODS

### 3.1 Criteria for considering studies for this review

#### 3.1.1 Types of studies

This systematic review shall include longitudinal studies that assess leadership styles as preceding measures of patient safety. Longitudinal studies that will be considered could either ideally have a full panel design, in which assessment of the leadership style temporarily precedes patient safety indicators but controlling for initial levels of the variables of interest, as well as studies of longitudinal nature in which patient safety measures are captured at a later point in time than leadership styles. We will focus on patient safety indicators measured in hospital care. Any form of cross‐sectional study is excluded from this review, as well as studies that do not include both leadership styles and patient safety indicators. Moreover, we will be focusing only on empirical papers because the emphasis of the systematic review is to understand the magnitude of the relationship. Therefore papers of a theoretical nature and reviews will be excluded. Included studies will use controls for hospital size, patient demographic characteristics, availability of human and physical resources, as well as skills and knowledge of patient safety preparedness. The choice of controls will be determined in accordance with guidelines provided by Spector and Brannick (2011). Studies that consist of randomized controlled trials will also be included if found.

#### 3.1.2 Types of participants

The studies that will be included in this review will be limited to those set in hospital settings across the continuum of acute to chronic care that deliver services to patients and run by hospital staff (administrative, technical and medical personnel) of any age group. All other healthcare settings that do not fit this definition such as nursing elderly people homes will be excluded.

#### 3.1.3 Types of interventions

We will consider different leadership styles assessed solely through followers’ perceptions or external raters’ evaluation. The literature mentions various leadership styles within hospitals such as transformational, authentic, distributed, and adaptive leadership. Nevertheless, other styles that are perceived by followers or by independent raters may emerge in the search. These will be included in the systematic review for use in our analysis and synthesis of research results.

#### 3.1.4 Types of outcome measures

**Figure 1:**
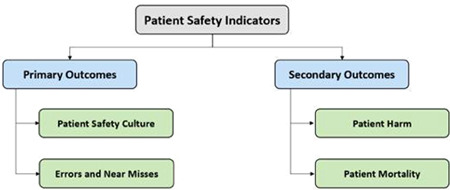
Patient safety indicators: Primary and secondary outcomes.

We will classify patient safety indicators into primary and secondary outcomes (as illustrated in Figure 1). All outcomes will be categorized according to their time frame (6 months, 1 year, 2–3 years, 4+ years).

##### Primary outcomes

The primary outcomes of this systematic review are patient safety climate and errors/near misses.

The IOM (Institute of Medicine, 2000; Institute of Medicine, 2001) refers to safety as an attribute of quality of care, with other dimensions namely efficiency, effectiveness, timeliness, equity and patient centeredness. Indeed, a systematic review by Sfantou et al. (2017) is entitled “Importance of Leadership Style towards Quality of Care Measures in Healthcare Settings: A Systematic Review.” The contribution of our systematic review is to focus specifically on patient safety rather than on the entire wider spectrum of quality of care.

Patient safety culture is a multidimensional concept. The most commonly reported dimensions include: “Communication openness,” “Feedback and communication about error,” “Teamwork within units,” “Teamwork across units,” “Non‐punitive response to error,” “Organizational learning‐continuous improvement,” “Supervisor/manager expectations and actions promoting patient safety,” “Staffing,” “Handoffs and transitions,” and “Management support for patient safety” (Hellings et al., 2007, Reis et al., 2018). In addition, a literature review by Sammer et al. (2010) reported that the dimensions of hospital culture related to patient safety can be organized into seven subcultures: “Teamwork,” “Evidence‐based,” “Leadership,” “Communication,” “Learning,” “Just,” and “Patient‐Centered.” Patient safety culture can be measured through surveys or questionnaires administered to hospital staff.

Medical errors are “the failure of a planned action to be completed as intended or the use of a wrong plan to achieve an aim” (Institute of Medicine, 2000). In health care, high error rates may bear severe consequences, usually related to intensive care, operating rooms and emergency units (Baker et al., 2004). In the United States alone, up to 98,000 patients die and more than 1 million are injured each year as a result of preventable medical errors (Kohn, 1999). Approximately 80 percent of medical errors are system‐derived (Leonard et al., 2004). Leaders across health care systems have legal, as well as moral and ethical obligations to ensure and maintain high quality of care and safety of patients, who should always be their highest priorities (Parand et al., 2014). However, our observations suggest that research and policy analysis of patient safety indicators is rather selective and fragmented. Indeed, health care systems are prone to error, and the risk of adverse events is significant (De Vries et al., 2008; Kohn, 1999). Measures that capture errors and near misses will be categorized as general (we may only find perceptions of professionals committing non‐specified errors/near misses), human induced (e.g., prescribing/medication errors, infection control behavior, professional task errors, iatrogenic conditions) and process defects (e.g., person identification, surgical procedures, communication errors—human or virtual). Errors will be classified according to the National Coordinating Council for Medication Error Reporting and Prevention (MERP, 2001) index, originally developed for medication errors (National Coordinating Council for Medication Error Reporting and Prevention, 2001), as: (i) No error; (ii) Error, no harm.

##### Secondary outcomes

The secondary outcomes of this systematic review are patient harm and mortality.

Harm has been defined by the National Coordinating Council for Medication Error Reporting and Prevention (NCC MERP) as “Impairment of the physical, emotional, or psychological function or structure of the body and/or pain resulting therefrom.” (MERP, 2001). With regard to mortality, we will consider iatrogenic mortality, which is to say that patient death occurs as a result of a medical error. Secondary outcomes will be classified according to the NCC MERP index as: i) Error, harm; ii) Error, death.

### 3.2 Search methods for identification of studies

We will develop our search strategy to investigate the prediction of hospital leadership styles on patient safety indicators. Studies reported in peer‐review journals (based on both theoretical and empirical research) will be included. There will be no language restriction, whereas English shall remain a key working language. Studies published in other languages shall be involved as long as our team can handle effective translation and/or external support can be provided. Papers will be focused on the following topics: (i) leadership and leadership styles; (ii) patient safety and (iii) patient safety indicators. Papers not providing evidence on these areas will be excluded. Since the concept of patient safety dates back to 1964 with the awareness of the hazards of hospitalization (Schimmel, 1964), our systematic review will consider studies published from 1964 onwards. Therefore, our search strategy will be limited by the following parameters: studies focused on leadership styles; studies evaluating patient safety indicators; and studies published from 1964 onwards.

#### 3.2.1 Electronic searches

This search will be conducted using keywords and Medical Subject Headings (MeSH) terms in the following databases: ABI/INFORM Collection (ProQuest); Business Market Research Collection (ProQuest); CINAHL Complete (EBSCO); Cochrane Central Register of Controlled Trials (EBSCO); Directory of Open Access Journals (DOAJ); EMBASE; Emerald Insight; Medline (Ovid); Scopus; and Web of Science Core Collection. The choice of these databases is due to their coverage on leadership studies and hospital research. The search strategy developed for Medline (Ovid) is presented in Supporting Information: Appendix 1, and it will be adapted for the other databases.

#### 3.2.2 Searching other resources

Hand‐searches, gray literature searches, website searches, and reference harvesting will be performed (looking for references within known studies to find additional material). In addition to papers retrieved according to the search strategy as presented above, articles and studies selected according to the authors’ personal knowledge and background might also be included—if pertinent to the research questions and scope of this review.

Hand search: Hand search will be conducted for the following relevant journals publishing in the area to locate articles not emerging from database search:

1. American Psychological Association Journals Special Issues on Leadership: https://www.apa.org/pubs/journals/special/4016201
2. Academy of Management Journals: https://journals.aom.org/action/doSearch?AllField=leadership
3. International Leadership Association Journals: http://ila-net.org/Publications/Journals.htm

Furthermore, we will consult and evaluate reference lists within retrieved articles. Gray literature: Gray literature will also be included if relevant to our research questions and if fulfilling the inclusion criteria. In this regard, we will search for the following items:

1. Conference proceedings from the American Psychological Association and Society for Social Work and Research
2. Publications from the UK King's Fun d organization (https://www.kingsfund.org.uk/publications)
3. Publications from the Patient‐Centered Outcomes Research Institute (PCORI) (https://www.pcori.org/research-results/pcori-literature)
4. MedEdPortal (https://www.mededportal.org/)

Personal communication: We will communicate with leading experts on hospital leadership through personal discussions, emails and requests for information to help us retrieve any missing material from our searches. We will also contact leading experts who run courses on hospital leadership at Universities. Communications will also be sought with Campbell's team to seek support whenever the need arises. A call for eligible studies will be posted on the websites of the American Academy of Management (AOM), divisions Health Care management (HCM), Organizational Behavior (OB) and Human Resources Management (HRM); European Health Management Association (EHMA); European Hospital Management Association (EHMA); European Association of Hospital Managers (EAHM); European Hospital and Healthcare Federation (HOPE); Australian and New Zealand Academy of Management (ANZAM); Asian Academy of Management (AAOM); South Asian Academy of Management (SAAM). All personal contacts will be documented.

### 3.3 Data collection and analysis

#### 3.3.1 Selection of studies

Citations identified from electronic databases searches will be imported in a referencing software and duplicates will be removed. Two authors will independently screen all titles and abstracts according to the inclusion/exclusion criteria. We will retrieve the full texts of all citations that are deemed eligible by at least one of the two authors. Full texts will be assessed by the same two authors independently, to determine eligibility based on the inclusion/exclusion criteria, with disagreement solved through discussion and with involvement of a third author, if necessary. Reasons for exclusion will be documented. Some studies might have been reported in multiple documents, and we aim to identify all documents associated with each included study. The different phases of study selection will be identified and illustrated according to the Preferred Reporting Items for Systematic Reviews and Meta‐Analyses (PRISMA) flow diagram. This diagram will also include the final number of studies included, if any. A screening guide is reported in Supporting Information: Appendix 2.

#### 3.3.2 Data extraction and management

Included studies will be reviewed by two authors, who will perform data extraction independently, with disagreement solved through discussion and with involvement of a third author, if necessary. The data extraction form will include the following items: article number; reference; year; author(s); title; country, health service; setting; aim(s)/objective(s); research design; method; population/sample; findings (effect size, in terms of number of participants with events and total number of participants—if dichotomous outcomes; mean, standard deviation and number of participants—if continuous outcomes); and outcome (primary and/or secondary). A draft of the extraction form is reported in Supporting Information: Appendix 3. Data will be collected into a Microsoft Excel spreadsheet.

#### 3.3.3 Assessment of risk of bias in included studies

**Figure 2:**
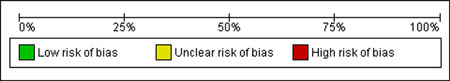
The level of risk of bias.

Each study will be reviewed by at least two interdependent raters based on pre‐defined criteria and any disagreements will be discussed with a third person from the team. Disagreements will be reconciled and recorded. Risk of bias will be assessed using the Cochrane risk‐of‐bias tool for randomized trials version 2 (RoB 2) for randomized controlled trials (Sterne et al., 2016) through the specific RoB 2 Excel tool (available at https://www.riskofbias.info/welcome/rob-2-0-tool/current-version-of-rob-2), or the Risk Of Bias In Non‐Randomized Studies of Interventions (ROBINS‐I) for non‐randomizes studies. The level of risk will be gauged according to Figure 2.

#### 3.3.4 Measures of treatment effect

If the outcomes of interest are expressed as dichotomous, we will summarize the results as risk ratio (RR, 95% CI). If the outcomes are continuous we will adopt the “r” as our indicator of treatment effect. We will consult with Campbell statisticians for analyses when considering the combination of different measures of treatment effects (risk ratio and “r”).

#### 3.3.5 Unit of analysis issues

Two types of unit of analysis problem might arise in our systematic review. First, if we encounter clustered studies that do not provide appropriate clustered analysis of data, we will use the corrections recommended in the Cochrane handbook. Second, some studies may include more than two leadership styles. For example, a study might report outcomes associated with four different leadership styles (four “arms”). If we find multiple‐armed studies, we will do pair‐wise comparisons of relevant leadership‐style groups.

#### 3.3.6 Dealing with missing data

Missing data could refer to hospital or leader characteristics, statistics (for analyzing effect sizes), cases, outcome measures, or endpoints. Should we find missing data in retrieved papers or reports we will personally contact the authors in the attempt to collect data. We will assess missing data as part of risk of bias during the study evaluation and will address them by adopting appropriate strategies.

#### 3.3.7 Assessment of heterogeneity

Heterogeneity among included studies could arise from several elements, including different populations, contexts, study design, outcome measures. It will be assessed using the *χ* ^2^ test of heterogeneity and the *I* ^2^ statistics, provided by RevMan. We will also assess the degree of overlap of the confidence intervals in the forest plots. In the predictive syntheses, we will capture the diversity of models tested, as in the case of regression analyses with multiple covariates. Differences in the models tested could be a factor in heterogeneity of findings and effects.

#### 3.3.8 Assessment of reporting biases

Publication bias will be assessed through the creation of funnel plots and associated statistical methods, if there are at least 10 included studies measuring the same outcome. We will also consult with a Campbell methodologist for these analyses.

#### 3.3.9 Data synthesis

We will perform a random‐effect meta‐analysis, since heterogeneity in this field is likely to be high. The inverse variance method will be used to give weight to each study. We will conduct pair‐wise meta‐analysis using the software Review Manager (RevMan).

#### 3.3.10 Subgroup analysis and investigation of heterogeneity

For the purposes of this review, we will perform moderator analysis to assess whether the relationships between leadership style and outcomes shows any differences by the leaders’ role in a hierarchy (defined as supervisors, middle managers, senior managers, according to Flin and Yule (2004). We will perform moderator analysis using STATA if there are more than 20 studies. We will consult with Campbell statisticians about the best methods for moderator analysis.

#### 3.3.11 Sensitivity analysis

We will perform sensitivity analysis to assess the potential impact of outliers and the potential impact of the risk of bias. Furthermore, we will use sensitivity analysis to consider the potential impact of any deviations from this original protocol (e.g. changes in the inclusion/exclusion criteria).

#### 3.3.12 Summary of findings and assessment of the certainty of the evidence

Summary of findings and assessment of the certainty of the evidence.

## CONTRIBUTIONS OF AUTHORS

See Table 1.

**Table 1:** Roles and responsibilities of authors.

Team member	Content	Systematic Review methods	Statistical analysis	Information retrieval
Sandra C. Buttigieg	L	L	L	C
Nicoletta Riva	S	S	S	S
Gianpaolo Tomaselli	S	S	S	S
Emanuel Said	S	S	S	S
Elaine Grech	S	S	S	S
Vincent Cassar	L	L	L	C

*Note*: Note, if the protocol or review is not submitted within 6 months and 18 months of title registration, respectively, the review area is opened up for other authors. Date for draft protocol submission: September 30, 2019. Date for draft review submission: July 31, 2020. Sandra C. Buttigieg will be responsible for updating this review should any new developments arise and in accordance with Campbell Collaboration guidelines and as funding becomes available.

Abbreviations: C, must be consulted; L, leading role; S, supportive role.

## DECLARATIONS OF INTEREST

None.

## SOURCES OF SUPPORT

**Internal sources**

- No sources of support provided
**External sources**
- No sources of support provided

## Supporting information

Supporting information.
