# Supplementary material for: PROTOCOL: Do hospital leadership styles predict patient safety indicators? A systematic review
Source: Campbell Syst Rev. 2023 Jul 7;19(3):e1338. doi: 10.1002/cl2.1338 (PMC10327627; doi:10.1002/cl2.1338)
Supplement: Supplementary file 1 — Supporting information. [file CL2-19-e1338-s001.docx]

# Appendices

## 1 Ovid MEDLINE search strategy

| 1 | exp Leadership/ |
| --- | --- |
| 2 | (leader* or manager* or chief* or supervisor*).mp. |
| 3 | 1 or 2 |
| 4 | exp Hospitals/ |
| 5 | (hospital* or "secondary care" or "tertiary care" or "acute care").mp. |
| 6 | 4 or 5 |
| 7 | exp Safety/ or safety.mp. |
| 8 | exp Medical Errors/ or exp Medication Errors/ or exp Diagnostic Errors/ |
| 9 | exp Near Miss, Healthcare/ |
| 10 | (error* or "near miss*").mp. |
| 11 | defect*.mp. |
| 12 | harm.mp. |
| 13 | adverse event*.mp. |
| 14 | exp Iatrogenic Disease/ or iatrogenic.mp |
| 15 | mortality.mp. or exp Mortality/ |
| 16 | 7 or 8 or 9 or 10 or 11 or 12 or 13 or 14 or 15 |
| 17 | 3 and 6 and 16 |
| 18 | limit 17 to yr="1964 -Current" |

## 2 Screening form

a) Does the document report a longitudinal study?

- Yes
- Unclear
- No – EXCLUDE

b) Was the study conducted in hospital setting?

- Yes
- Unclear
- No – EXCLUDE

c) Did the study evaluate a leadership style as type of intervention?

- Yes
- Unclear
- No – EXCLUDE

d) Did the study evaluate patient safety indicators?

- Yes
- Unclear
- No – EXCLUDE

## 3 Data Extraction Form

- Study ID:
- Full reference:
- First author surname (publication year):

**Study description**

- Type of publication:
  - peer-reviewed journal article
  - conference paper
  - technical report
  - other (please specify)
- Country:
- Inclusion criteria:
- Exclusion criteria:
- Funding source (if any):
- Study design:
  - Randomized controlled trial
  - Prospective cohort study
  - Retrospective cohort study
- Type of statistical analysis:

**Population characteristics**

- Type of health service:
- Population characteristics:
- Sample size:

**Intervention characteristics**

- Type of leadership:

**Outcome characteristics**

- Safety indicators evaluated and effect size:
  - Patient safety culture
  - Human induced errors (e.g. medication errors, infection control behaviour, professional task specific errors, iatrogenic conditions)
  - Process defects (e.g. person identification, surgical procedural errors, communication)
  - Patient harm
  - Patient mortality

**Risk of bias assessment**

**a) Randomized trials (adapted from the Cochrane risk-of-bias tool for randomized trials version 2**

- Bias arising from the randomisation process
  - Low risk of bias
  - Some concerns
  - High risk of bias
  - No information
- Bias due to deviations from intended interventions (effect of adhering to intervention)
  - Low risk of bias
  - Some concerns
  - High risk of bias
  - No information
- Bias due to missing outcome data
  - Low risk of bias
  - Some concerns
  - High risk of bias
  - No information
- Bias in measurement of the outcome
  - Low risk of bias
  - Some concerns
  - High risk of bias
  - No information
- Bias in selection of the reported result
  - Low risk of bias
  - Some concerns
  - High risk of bias
  - No information
- Overall risk of bias
  - Low risk of bias [low risk of bias for all domains]
  - Some concerns
  - High risk of bias [high risk of bias in at least one domain, or some concerns for multiple domains]

**b) Non-randomized trials (adapted from the Risk Of Bias In Non-Randomized Studies of Interventions (ROBINS-I); Sterne et al, 2016)**

- Bias due to confounding
  - Low risk of bias
  - Moderate risk of bias
  - Serious risk of bias
  - Critical risk of bias
  - No information
- Bias in selection of participants into the study
  - Low risk of bias
  - Moderate risk of bias
  - Serious risk of bias
  - Critical risk of bias
  - No information
- Bias in classification of interventions
  - Low risk of bias
  - Moderate risk of bias
  - Serious risk of bias
  - Critical risk of bias
  - No information
- Bias due to deviations from intended interventions
  - Low risk of bias
  - Moderate risk of bias
  - Serious risk of bias
  - Critical risk of bias
  - No information
- Bias due to missing data
  - Low risk of bias
  - Moderate risk of bias
  - Serious risk of bias
  - Critical risk of bias
  - No information
- Bias in measurement of outcomes
  - Low risk of bias
  - Moderate risk of bias
  - Serious risk of bias
  - Critical risk of bias
  - No information
- Bias in selection of the reported result
  - Low risk of bias
  - Moderate risk of bias
  - Serious risk of bias
  - Critical risk of bias
  - No information
- Overall risk of bias
  - Low risk of bias [low risk of bias for all domains]
  - Moderate risk of bias [low or moderate risk of bias for all domains]
  - Serious risk of bias [serious risk of bias in at least one domain]
  - Critical risk of bias [critical risk of bias in at least one domain]
